# A Novel Homozygous *COL4A3* c.1873G>A (p.Gly625Ser) Variant Presenting With Autosomal Recessive Alport Syndrome: Clinical, Genetic, and Segregation Evidence

**DOI:** 10.1016/j.ekir.2026.106528

**Published:** 2026-04-01

**Authors:** Zeynep Ural, Ülver Derici

**Affiliations:** 1 Department of Nephrology, Lösante Hospital, Ankara, Turkey; 2 Department of Nephrology, Faculty of Medicine, Gazi University, Ankara, Turkey

**To the Editor:**

We read with great interest the report by Solanki *et al.*,^1^ which effectively illustrates the diverse phenotypic expression among individuals with *COL4A3* variants and emphasizes the ongoing diagnostic challenges in type IV collagen–related diseases. Their findings highlight the persistent challenge of distinguishing hereditary basement membrane disorders within adult nephrology, particularly when biopsy findings resemble primary podocytopathies.^1^

In concordance with their observations, we describe a multigenerational Turkish family in which 2 siblings presented with biopsy-proven focal segmental glomerulosclerosis, progressive kidney dysfunction, and sensorineural hearing loss. Despite this classic syndromic constellation, both individuals were initially misclassified as having nongenetic focal segmental glomerulosclerosis because earlier-generation next-generation sequencing erroneously reported a heterozygous “nonpathogenic” finding, leading to prolonged immunosuppression, delayed genetic counseling, and missed opportunities for timely renal replacement planning.

Subsequent reevaluation using targeted clinical exome sequencing identified a previously undescribed *COL4A3* c.1873G>A (p.Gly625Ser) variant, which was confirmed by Sanger segregation analysis and classified as likely pathogenic (American College of Medical Genetics and Genomics, 2015), consistent with autosomal recessive Alport syndrome. Segregation demonstrated the expected pattern: homozygous in affected siblings, heterozygous in relatives with isolated hematuria, and wild type in unaffected family members (Figure 1). This variant represents a new addition to the mutational spectrum of *COL4A3*. Because glycine substitutions within the collagenous domain destabilize the triple helix, missense variants at these positions are strongly associated with pathogenicity. The homozygous presence of this variant in 2 affected siblings, its segregation with disease, and its absence in population databases together provide compelling evidence for its clinical significance.^2^, ^3^, ^4^

**Figure 1:**
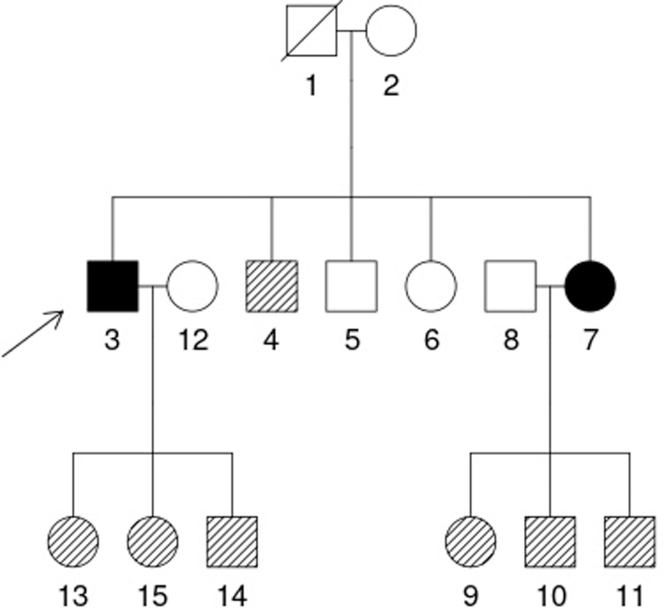
Family pedigree and segregation analysis of the *COL4A3* c.1873G>A (p.Gly625Ser) variant. The pedigree illustrates an autosomal recessive inheritance pattern. Males are shown as squares and females as circles. Fully shaded symbols represent affected individuals, whereas striped symbols indicate heterozygous carriers of the *COL4A3* variant. The proband is marked with an arrow.

This exceptional case underscores several critical diagnostic challenges raised by Solanki *et al.*^1^ First, the risk of misdiagnosing hereditary collagen IV–associated nephropathies as primary focal segmental glomerulosclerosis. Second, next-generation sequencing read-depth limitations remain a significant cause of misinterpretation. Finally, accurate molecular diagnosis directly influences living donor evaluation, preventing unsafe donation among obligate carriers.

Together, their report and our case highlight the urgent need to integrate genetic testing earlier in the evaluation of adults presenting with focal segmental glomerulosclerosis patterns, and to emphasize segregation analysis and Sanger confirmation whenever clinical suspicion remains high. These steps are essential to avoid misdiagnosis, reduce unnecessary immunosuppression, and enable appropriate donor selection in familial disease.

## Disclosure

All the authors declared no competing interests.

## Patient Consent

The patient gave informed consent to publish this case.
